# Chloroform in Catalepsy

**Published:** 1861-12

**Authors:** Ashbel Woodward

**Affiliations:** Franklin, Conn.


### ARTICLE VI.

### CHLOROFORM IN CATALEPSY.

By ASHBEL WOODWARD, M. D., of Franklin, Conn.

The patient, Miss E. W., seventeen years of age, was attacked early in August, 1861. The catamenia had been somewhat irregular, the general disturbance of the system being referred to a punctured wound of the foot.

Iron, guaiac, cimicifuga rac., and like remedies were administered without producing beneficial effects upon the general health.

At first, the paroxysms were slight in character, and of short duration, but gradually became more severe. The attacks occurred but once in twenty-four hours, invariably commencing about seven o'clock in the evening.

The periodical nature of the complaint suggested the use of quinine, which was given for two successive days, in doses sufficiently large to produce a decided impression. The remedy seemed to aggravate the severity of the paroxysm, and was accordingly discontinued.

At this juncture, I was induced to try chloroform, from having previously used it with great success in treating infantile convulsions. The patient at first took three doses, of twenty drops each, diluted in a wine-glass of water, at intervals of an hour, shortly before the time of the expected onset. No improvement was experienced on the first trial. The second day the dose was increased to twenty-five drops, and five doses were given at intervals of thirty minutes. The remedy so impressed the system, that on the third day from the beginning of the chloroform treatment, the paroxysm did not re-appear. From that time onward, her general health has gradually improved till now, November 16, it is excellent.

It should, perhaps, be stated, that the patient showed an extreme dislike to the medicine.

